# Binary Restructuring Particle Swarm Optimization and Its Application

**DOI:** 10.3390/biomimetics8020266

**Published:** 2023-06-17

**Authors:** Jian Zhu, Jianhua Liu, Yuxiang Chen, Xingsi Xue, Shuihua Sun

**Affiliations:** 1 School of Computer Science and Mathematics, Fujian University of Technology, Fuzhou 350118, China; jianzhu98@foxmail.com (J.Z.); 15060485887@163.com (Y.C.); jack8375@gmail.com (X.X.); shuihua.11109029@gmail.com (S.S.); 2 Fujian Provincial Key Laboratory of Big Data Mining and Applications, Fuzhou 350118, China

**Keywords:** particle swarm optimization, binary particle swarm optimization, restructuring particle swarm optimization, feature selection

## Abstract

Restructuring Particle Swarm Optimization (RPSO) algorithm has been developed as an intelligent approach based on the linear system theory of particle swarm optimization (PSO). It streamlines the flow of the PSO algorithm, specifically targeting continuous optimization problems. In order to adapt RPSO for solving discrete optimization problems, this paper proposes the binary Restructuring Particle Swarm Optimization (BRPSO) algorithm. Unlike other binary metaheuristic algorithms, BRPSO does not utilize the transfer function. The particle updating process in BRPSO relies solely on comparison results between values derived from the position updating formula and a random number. Additionally, a novel perturbation term is incorporated into the position updating formula of BRPSO. Notably, BRPSO requires fewer parameters and exhibits high exploration capability during the early stages. To evaluate the efficacy of BRPSO, comprehensive experiments are conducted by comparing it against four peer algorithms in the context of feature selection problems. The experimental results highlight the competitive nature of BRPSO in terms of both classification accuracy and the number of selected features.

## 1. Introduction

The Particle Swarm Optimization (PSO) algorithm, initially proposed by Eberhart and Kennedy in 1995 [1], is a heuristic search algorithm grounded in evolutionary algorithms and artificial life. PSO exhibits a memetic nature, facilitating the retention of well-performing particles. Moreover, the parameters of PSO can be easily adjusted, enabling its applicability to a wide range of practical problems. Drawing inspiration from the foraging behavior of birds, PSO utilizes historical experiences and social knowledge to explore the solution space and identify optimal solutions. Each particle in PSO possesses position and velocity vectors, with the former representing a potential solution and the latter governing adjustments to the particle’s flight direction. The updating formula for the position and velocity vectors in the canonical PSO [2] is provided below.

(1) $$v_{i}^{d}\left(t+1\right)=w\times v_{i}^{d}\left(t\right)+c_{1}\times r_{1}\times \left(pbest_{i}^{d}-x_{i}^{d}\left(t\right)\right)+c_{2}\times r_{2}\times \left(gbest^{d}-x_{i}^{d}\left(t\right)\right)$$

(2) $$x_{i}^{d}\left(t+1\right)=x_{i}^{d}\left(t\right)+v_{i}^{d}\left(t+1\right)$$

where *d* denotes the dimensionality of the problem. *i* represents the number of particles in the swarm. The iteration number during the evolutionary process is denoted by *t*. *w* is the inertial weight, which controls the proportion inherited from the previous velocities in updating current velocity. Two acceleration factors, $c_{1}$ and $c_{2}$ control the impact of particle learning pbest and gbest on velocity. Additionally, two random numbers, $r_{1}$ and $r_{2}$, uniformly distributed in the interval [0, 1], are utilized. The variable $pbest_{i}$ represents the historical optimal solution of the *i*th particle, while gbest represents the global optimal solution identified during the course of the evolutionary process.

While Particle Swarm Optimization (PSO) has predominantly been explored for optimization problems in continuous spaces, its application to optimization problems in binary spaces has received relatively limited attention. In 1997, Eberhart and Kennedy introduced a binary variant of PSO called Binary Particle Swarm Optimization (BPSO) specifically designed for addressing optimization problems in discrete binary spaces [3]. In BPSO, the position vector is encoded in a binary format. Building upon PSO, BPSO incorporates a sigmoid transfer function to map the velocity values onto the interval [0, 1]. The subsequent position value is determined by comparing the output of the transfer function to a randomly generated number within the [0, 1] interval, resulting in either “0” or “1”. The specific updating formula for BPSO is elucidated below.

(3) $$Tf\left(v_{i}^{d}\left(t+1\right)\right)=\mathrm{sigmoid}\left(v_{i}^{d}\left(t+1\right)\right)=\frac{1}{1+e^{-v_{i}^{d}\left(t+1\right)}}$$

(4) $$x_{i}^{d}\left(t+1\right)=\left\{\begin{array}{cc} 1 & \;\mathrm{if}\;Tf\left(v_{i}^{d}\left(t+1\right)\right)>\mathrm{rand}\left(\right)\\ 0 & \;\mathrm{if}\;Tf\left(v_{i}^{d}\left(t+1\right)\right)\leq \mathrm{rand}\left(\right) \end{array}\right.$$

Since the initial proposal of the BPSO algorithm, researchers have made notable advancements in its efficacy for solving diverse practical discrete combinatorial optimization problems [4,5,6]. Despite its merits, BPSO inherits certain limitations from the original PSO algorithm due to their shared velocity updating formula. Notably, BPSO exhibits a tendency to converge prematurely when confronted with complex problems. The performance of BPSO is significantly influenced by the design of the transfer function, and the sigmoid transfer function fails to adequately capture the characteristics of PSO. Consequently, researchers have sought to address these challenges by modifying the velocity updating formula or introducing alternative transfer functions. Numerous strategies have been employed and various new transfer functions were designed. While these strategies have enhanced the performance of BPSO in certain aspects, they have also introduced additional algorithmic complexity and increased computational overhead.

To address the prevailing challenges related to transfer functions and computational costs in existing algorithms, this study introduces a variant of Binary Particle Swarm Optimization called Binary Restructuring Particle Swarm Optimization (BRPSO). The BRPSO algorithm is developed based on the restructuring PSO (RPSO) framework proposed in 2022 [7]. By eliminating the use of transfer functions, BRPSO achieves a streamlined algorithmic flow and enhances computational efficiency.

The main contributions of this study can be summarized as follows.

- In this paper, a binary variant of the Restructuring Particle Swarm Optimization (RPSO) algorithm is proposed, aiming to extend the capabilities of RPSO towards addressing binary optimization problems.
- The proposed approach eliminates the incorporation of both the velocity updating formula and the transfer function commonly utilized in traditional BPSO. This removal serves to eliminate the influence of the transfer function and simplify the algorithmic flow.
- The introduction of a novel perturbation term in BRPSO presents an advancement that not only reduces the number of required parameters but also satisfies diverse requirements across different stages of evolution.

The remainder of this paper is structured as follows: Section 2 introduces the related work of this paper which includes feature selection and RPSO. The proposed binary RPSO is presented in Section 3. In Section 4, the experimental results and discussion are given. Lastly, concluding remarks are provided in Section 5.

## 2. Related Work

In this section, the development of BPSO will be reviewed first. Following this, the concept of feature selection is introduced as the test problem. Furthermore, the Restructuring Particle Swarm Optimization algorithm for continuous optimization problems is introduced at the end.

### 2.1. Review of BPSO

The improvement of BPSO can be broadly categorized into two avenues: the modification of the transfer function and the improved updating strategies in velocity.

#### 2.1.1. Reviews of Transfer Function

The original BPSO algorithm employs the sigmoid function as the transfer function for velocity transformation. However, this choice of transfer function does not align with the fundamental characteristics of PSO. In PSO, a large absolute value of velocity means the particle should take greater movement to search the optimal solution. Conversely, a small absolute velocity value indicates that particle has approached the optimal solution and needs small movement. Nevertheless, in the original BPSO algorithm, a negative large velocity is likely to result in a next position of 0, while a positive large velocity is prone to yield a next position of 1. Otherwise, the next position takes 0 or 1 with a probability 0.5 when the velocity takes 0.

In 2008, a probability binary PSO (PBPSO) algorithm was proposed by Wang et al. [8]. PBPSO incorporates a novel linear transfer function and a set of rules to generate new positions. Specifically, it utilizes the continuous search space position to update the binary position. The detailed updating rule is provided below.

(5) $$TF\left(x_{i}^{d}\left(t+1\right)\right)=\frac{\left(x_{i}^{d}\left(t+1\right)-R_{\min}\right)}{R_{\max}-R_{\min}}$$

(6) $$\begin{array}{cc} xb_{i}^{d}\left(t+1\right) & =\left\{\begin{array}{ccc} 1 & \;\mathrm{if}\; & \mathrm{rand}\left(\right)\leq \mathrm{TF}\left(x_{i}^{d}\left(t+1\right)\right)\\ 0 & \;\mathrm{if}\; & \mathrm{rand}\left(\right)>\mathrm{TF}\left(x_{i}^{d}\left(t+1\right)\right) \end{array}\right. \end{array}$$

where [*R*_min_, *R*_max_] is a predefined range to transfer continuous position into the probability value. However, the linear transfer function also faced the same drawbacks as S-shape transfer function. To overcome the shortcoming of inconsistency between S-shape transfer function and PSO characteristics, Nezamabadi-pour et al. [9] proposed a new discrete binary PSO (NBPSO) which used a new transfer function described as follows.

(7) $$TF\left(V_{i}^{d}\right)=\left|\tanh\left(\alpha v_{i}^{d}\right)\right|$$

(8) $$\begin{array}{cc} x_{i}^{d}\left(t+1\right) & =\left\{\begin{array}{cc} \;\mathrm{exchange}\;\left(x_{i}^{d}\left(t\right)\right) & \;\mathrm{if}\;TF\left(v_{i}^{d}\left(t+1\right)\right)>\mathrm{rand}\left(\right)\\ x_{i}^{d}\left(t\right) & \;\mathrm{if}\;TF\left(v_{i}^{d}\left(t+1\right)\right)\leq \mathrm{rand}\left(\right) \end{array}.\right. \end{array}$$

where α is a constant value. When the next position will change when the transfer value is greater than a random number in the range [0, 1]. On the contrary, it remains the same. The use of V-shape transfer function and new updating rules avoid the conflicts between the transfer function and PSO. However, the global exploration ability of NBPSO is still insufficient. When the position is a local optimal solution and the velocity approaches zero, the algorithm hardly escapes the local optimal space when using V-shape transfer function.

Mirjalili and Lewis [10] proposed six modified transfer functions and divided them into two categories: S-shape and V-shape. Through the comparison on 25 benchmark functions among six modified transfer functions, the results that the proposed V-shape transfer function performs better than the S-shape transfer function are obtained.

In order to address the limitations of existing transfer functions in providing adequate exploration and exploitation capabilities for BPSO, Md. Jakirul Islam et al. [11] conducted research on the exploration and exploitation stages of BPSO, as well as analyzed the behavior of existing S-shape, V-shape, and linear transfer functions. They raised a time-varying transfer function which provided a high flipping probability of particles in the early stages to ensure the exploration of search space and a low flipping probability in the final stages to exploit the solution space. However, it faced the same drawbacks as the traditional S-shape transfer function.

Different shape transfer function gives BPSO distinct search ability, so Mirjalili et al. [12] proposed a novel U-shape transfer function with multiple alterable parameters, and applied it in BPSO. Through the experiments in a set of benchmark functions and 0/1 knapsack problems, the following U-shape transfer function had been proved performances better than S-shape and V-shape transfer functions.

(9) $$TF\left(V_{i}^{d}\right)=\alpha \left|x^{\beta }\right|$$

where α and β are two parameters that control the slope and width of the transfer function. However, U-shape transfer function has the similar disadvantage as V-shape transfer function.

In order to address the issue of BPSO algorithms being prone to local optima, Guo et al. [13] proposed an asymmetric mapping function named Z-shape transfer function to map the continuous search space to a binary space. Nine typical benchmark functions are adopted to test the effectiveness of Z-shape, S-shape, and V-shape transfer functions. Furthermore, the raised Z-shape transfer function achieved the best performance. The Z-shape transfer function is defined as follows.

(10) $$TF\left(v_{i}^{d}\left(t\right)\right)=\sqrt{1-a^{v_{i}^{d}\left(t\right)}}$$

where α is the parameter to control the slope of the transfer function. Because BPSO easily stuck into the local optimal solution or converged at a slower speed when using the exitied transfer function, Beheshti [14] raised a novel X-shape transfer function to enhance the exploration and exploitation ability of BPSO (XBPSO) in the discrete binary search space.

(11) $$TF_{1}\left(v_{i}^{d}\left(t+1\right)\right)=\frac{-v_{i}^{d}\left(t+1\right)}{1+\left|-v_{i}^{d}\left(t+1\right)\right|\times 0.5}+0.5$$

(12) $$\begin{array}{cc} y_{i}^{d} & =\left\{\begin{array}{cc} 1 & \;\mathrm{if}\;{\mathrm{rand}}_{1}>TF_{1}\left(v_{i}^{d}\left(t+1\right)\right)\\ 0 & \;\mathrm{if}\;{\mathrm{rand}}_{1}\leq TF_{1}\left(v_{i}^{d}\left(t+1\right)\right) \end{array}\right. \end{array}$$

(13) $$TF_{2}\left(v_{i}^{d}\left(t+1\right)\right)=\frac{v_{i}^{d}\left(t+1\right)-1}{1+\left|v_{i}^{d}\left(t+1\right)-1\right|*0.5}+0.5$$

(14) $$\begin{array}{cc} z_{i}^{d} & =\left\{\begin{array}{cc} 1 & \;\mathrm{if}\;{\mathrm{rand}}_{2}<TF_{2}\left(v_{i}^{d}\left(t+1\right)\right)\\ 0 & \;\mathrm{if}\;{\mathrm{rand}}_{2}\geq TF_{2}\left(v_{i}^{d}\left(t+1\right)\right) \end{array}\right. \end{array}$$

(15) $$\begin{array}{cc} P_{i}\left(t+1\right) & =\left\{\begin{array}{cc} y_{i} & \;\mathrm{if}\;f\left(y_{i}\right)\;\mathrm{is}\;\mathrm{better}\;\mathrm{than}\;f\left(z_{i}\right)\\ z_{i} & \;\mathrm{if}\;f\left(z_{i}\right)\;\mathrm{is}\;\mathrm{better}\;\mathrm{than}\;f\left(y_{i}\right) \end{array}\right. \end{array}$$

In XBPSO, two positions $y_{i}$ and $z_{i}$ were first generated by two transfer functions. Furthermore, the better one is selected as $p_{i}\left(t+1\right)$. If the fitness value of $p_{i}\left(t+1\right)$ is better than the $x_{i}\left(t\right)$, $x_{i}\left(t+1\right)$ will be $p_{i}\left(t+1\right)$. Otherwise, XBPSO will generate two new positions by crossing operate *p_i_*(*t* + 1) and $x_{i}\left(t\right)$, and select the better one to be $x_{i}\left(t+1\right)$. Through the experiments, the effectiveness of the XBPSO had been demonstrated. However, it should be noted that XBPSO used at least twice the number of function evaluations in a generation. Furthermore, the computation cost of XBPSO is large. In the same year, Beheshti [15] proposed an upgrade transfer function (UTF) and applied it in BPSO. UTF also generates at least twice the number of solutions in each generation. Multiple fitness function evaluation times will be used in one generation in both X-shape and UTF.

Various new transfer functions were proposed in recent years, but they all exhibit different shortcomings. S-shape and linear-shape transfer functions do not fit the characteristics of the algorithm well. V-shape and U-shape transfer functions do not help the algorithm escape from the local optimal solution when the velocity approaches toward 0. X-shape and UTF require several fitness evaluations in one iteration.

#### 2.1.2. Reviews of Improvement in Updating Strategies of BPSO

The improvement in particle updating strategies focuses on parameter control and the modified learning models.

Liu et al. [16] used a mathematical model to analyze the role inertial weight was played in BPSO. Furthermore, obtained the conclusion that, when two acceleration factors are set to constant, a small inertia weight provides better exploration capability and a large inertia weight enhances exploitation. Furthermore, they proposed linearly increasing inertia weight BPSO(LIWBPSO), the setting of inertial weight is described as follows.

(16) $$\begin{array}{cc} w & =\left\{\begin{array}{cc} w+\frac{\pi \times (\bar{w}-\underline{w})}{\rho \times \bar{\pi }}, & \;\mathrm{if}\;\pi \leq \rho \times \bar{\pi }\\ \bar{w} & \;\mathrm{if}\;\rho \times \bar{\pi }<\pi \leq \bar{\pi } \end{array}\right. \end{array}$$

where π and $\bar{\pi }$ are the number of current iteration and the maximal number of iterations, respectively. *w* and $\bar{w}$ stand for the lower and upper bounds of *w*. ρ is a parameter that controls the increasing rate of *w*. The experiments adopted on 0–1 knapsack problem demonstrate the effectiveness of the raised increasing inertia weight. However, this research improved BPSO by only considering the influence of inertial weight.

Aiming to cope with premature convergence of the BPSO, Vieira et al. [17] put forward a modified BPSO (MBPSO) which used the mutation mechanism to avoid premature convergence of BPSO. Furthermore, Mingo López et al. [18] raised a hybrid genetic inspired BPSO (HPSOGO) which introduced crossover and mutation operations into BPSO. The personal historical best position and the global best position are used to crossover/mutation with the current position to generate the next position in HPSOGO.

Based on bare bones particle swarm optimization [19], Zhang et al. [20] first proposed the binary bare bones particle swarm optimization (BBPSO) and used it to solve the feature selection problem. BBPSO used a reinforced memory strategy to update the personal best historical position archive and the Uniform combination was adopted to balance the exploration and exploitation of BBPSO. In order to enable BPSO to better solve feature selection problems, Song et al. [21] raised a novel BBPSO with mutual information (MIBBPSO). In MIBBPSO, the correlation between features and class labels is used to initial the position to improve the convergence rate of algorithms. The supplementary operator and the deletion operator are designed to enhance the local search ability. An adaptive flip mutation operator is developed to balance the global search and local development in MIBBPSO.

A hybrid Taguchi binary particle swarm optimization (HTBPSO) was proposed by Jia et al. [22]. HTBPSO used the Taguchi method to enhance the local exploitation of BPSO and used catfish effect operation in the last 10% worse particle positions to avoid premature convergence.

Ji et al. [23] introduced three novel factors to improve BPSO (IBPSO) based on Lévy flight, weighting inertia coefficient, and mutation mechanism, respectively. New factors improve the performance of the algorithm in exploitation, exploration, and maintaining population diversity.

Hu et al. [24] raised a multi-surrogate assisted BPSO(MSABPSO) which used the multi-surrogate multi-swarm model to improve the convergence ability of binary PSO. Furthermore, a new dynamic S-shape transfer function was adopted to balance the abilities of exportation and exploitation for the MSABPSO. The experimental results on benchmark functions and feature selection problems showed that MSABPSO significantly improved prediction accuracy. However, it incurred greater costs associated with computational resources.

Among the literature mentioned above, various strategies were employed to enhance some capabilities of BPSO. However, there are some aspects they unconsidered. Some improvement strategies only considered the effect of a single variable. Some strategies combined with the other evolutional algorithms, increased the algorithm complexity and computation cost. Moreover, the use of some problem-specific improvement strategies limited the scalability of the algorithm to solve other problems.

### 2.2. Feature Selection

Feature selection is a data pre-processing strategy. It is a technique for extracting key features from data where it performs a series of complex calculations on a dataset to achieve the effect of eliminating irrelevant, redundant, and misleading features. Feature selection aims to decrease the number of features, which consequently results in a reduction in the duration of model training and computational expenses. Furthermore, it can enhance the performance of the model. Feature selection is widely used in pattern recognition and machine learning [25]. Feature selection can be roughly categorized into three groups based on the strategies employed: filter, wrapper and embedded [26]. The embedded methods embed feature selection into the learner training process. The filter techniques select relevant features purely based on the inherent relationships among the features, without requiring the integration of any learning method [27,28]. The Wrapper methods combine a search strategy with a learning method to identify the optimal subset of features for classification performance by utilizing a subset of features obtained through the search strategy to train a classifier [29,30]. The use of classifiers to train and evaluate each feature subset results in high computational overhead for the wrapper method. However, in the wrapper method, the classifier performance is directly used to evaluate the importance of features, which allows the wrapper method to achieve better classification performance than the filter technique. Due to the simplified implementation and low computation cost, BPSO is popularly used in the wrapper method. BPSO-based wrapper achieved significant performance in feature selection problems [31,32,33,34].

### 2.3. Restructuring Particle Swarm Optimization

Based on the theory analysis results of PSO which used linear system theory, Zhu et al. [24] proposed a novel structure of PSO named Restructuring Particle Swarm Optimization algorithm (RPSO). RPSO used only one formula to update the particles and took fewer parameters which simplified the algorithm. The specific formula is described as Equation (17)

(17) $$\begin{array}{c} x_{i}^{t}=r\times \;pbest_{i}+\left(1-r\right)\times \;\mathrm{gbest}\;+\left(-c\times \;\mathrm{rand}\;\right)\times \left(0.8\mathrm{rand}+w\right)\left(1-t/T\right)\\ +\left(c\times \;\mathrm{rand}\;\right)\times \left(0.8\mathrm{rand}+w\right)\left(1-t/T\right) \end{array}$$

where *x* represents the position of particle *i* in iteration *t*. *r* takes random number between 0 and 1. *w* linear decreasing from 0.8 to 0.2 with iterations. *t* is current iteration and *T* marks the maximum iteration.

Compared to the canonical PSO, RPSO has a simpler structure and fewer parameters. However, RPSO is proposed to solve the continuous optimization problem. There is no literature to use the BPSO to solve problems with binary variables. The setting of perturbation also takes many parameters, which seems complex.

## 3. Proposed Approach

In this section, the motivation behind our study is presented, followed by a detailed description of our approach and its corresponding flowchart. Finally, an explanation of the perturbation item’s configuration is provided.

### 3.1. Motivation

In recent years, numerous improved BPSO algorithms have been proposed, with a prevalent approach being the utilization of transfer functions to map velocity values onto the [0, 1] interval. Previous studies employed probability-based particle updates, guided by the mapped velocity values. The use of the transfer function complicated the algorithm flow. Moreover, the design of the transfer function plays a crucial role in determining the algorithm’s performance. A well-designed transfer function, aligned with the inherent characteristics of the algorithm, can enhance its performance. Conversely, an ill-suited transfer function can significantly impair the algorithm’s effectiveness. The transfer functions currently in use exhibit certain shortcomings, as discussed in Section 2.1.1. Furthermore, the improvement strategies presented in Section 2.1.2 encounter challenges related to the increased complexity of the algorithm.

In order to mitigate the impact of the transfer function and streamline the algorithmic process, this study introduces a binary variant of RPSO (BRPSO) that eliminates the use of a transfer function. The primary objective of BRPSO is to address binary optimization problems.

### 3.2. Binary Restructuring Particle Swarm Optimization

While RPSO is designed to tackle continuous optimization problems, binary optimization problems possess distinct solution spaces. To address this disparity, the Binary Restructuring Particle Swarm Optimization (BRPSO) is specifically developed for binary optimization problems. In this subsection, BRPSO is presented. It is a proposed method for addressing binary optimization problems.

In RPSO, the particle updating process is governed by a single formula influenced by the cumulative weights of the personal best (pbest) and global best (gbest) solutions, along with a perturbation term that diminishes with each iteration. Building upon these findings, the position update equation in BRPSO is expressed as Equation (18).

(18) $$x_{i}=r_{1}\times \;pbest_{i}+\left(1-r_{1}\right)\times \;\mathrm{gbest}\;+p$$

(19) $$\left\{\begin{array}{cc} x_{i}=1, & \;\mathrm{if}\;x_{i}>\;\mathrm{rand}\;\\ x_{i}=0, & \;\mathrm{if}\;x_{i}\leq \;\mathrm{rand}\; \end{array}\right.$$

where $r_{1}$ takes a random number in [0, 1] interval and changes with iteration. $pbest_{i}$ is *i*-th particle own best historical position and gbest is the global best position. *p* represents a decreasing perturbation term that eventually converges to 0. The detailed formula of *p* will propose in the next subsection. With the iteration process, the value obtained by Equation (18) will converge from [−1, 2] interval to [0, 1] interval which has the same expected value of [0, 1] interval. So, the final value of $x_{i}$ is “0” or “1” in each iteration is determined by comparing it with a random number in [0, 1] interval. Above updating formula can good substitutes the role of the transfer function.

**Figure 1:**
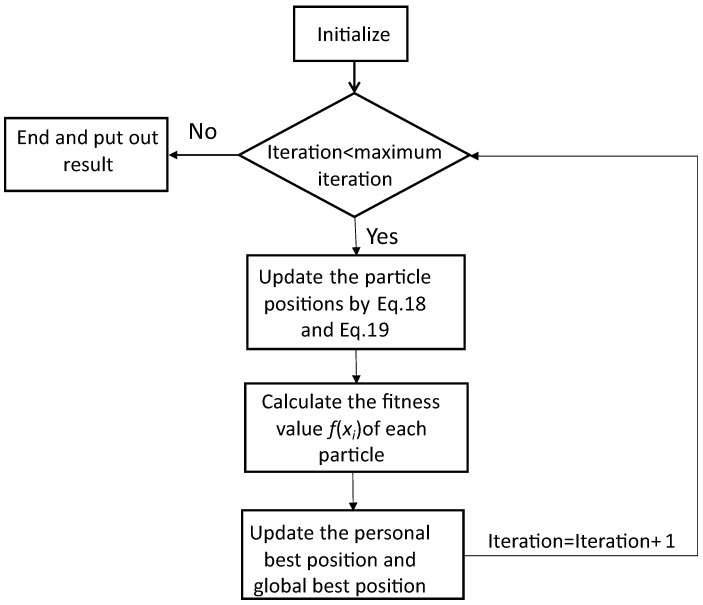
The flowchart of BRPSO.

The pseudocode of BRPSO framework is provided in Algorithm 1 and the flowchart is shown in Figure 1.
**Algorithm 1** BRPSO**Require:** maximum iteration, population size, dimension
1:**for** each particle *i* **do**2:    Initialize position $x_{i}$ for particle *i*;3:    Evaluate $f(x_{i})$ of particle *i* and set $p_{i}=x_{i}$;4:**end for**5:$p_{g}=min\left(p_{i}\right)$6:**while** iteration < Maximum iteration **do**7:    **for** i=1 to *N* **do**8:        Update position of particle *i* with Equations (18) and (19)9:        Evaluate particle *i*10:        **if** $f\left(x_{i}\right)<f\left(p_{i}\right)$ **then**11:           $p_{i}=x_{i}$12:        **end if**13:        **if** $f\left(x_{i}\right)<fit\left(p_{g}\right)$ **then**14:           $p_{g}=p_{i}$15:        **end if**16:    **end for**17:    iteration=iteration+118:**end while**
**Ensure:** $p_{g}$


### 3.3. Perturbation p

**Figure 2:**
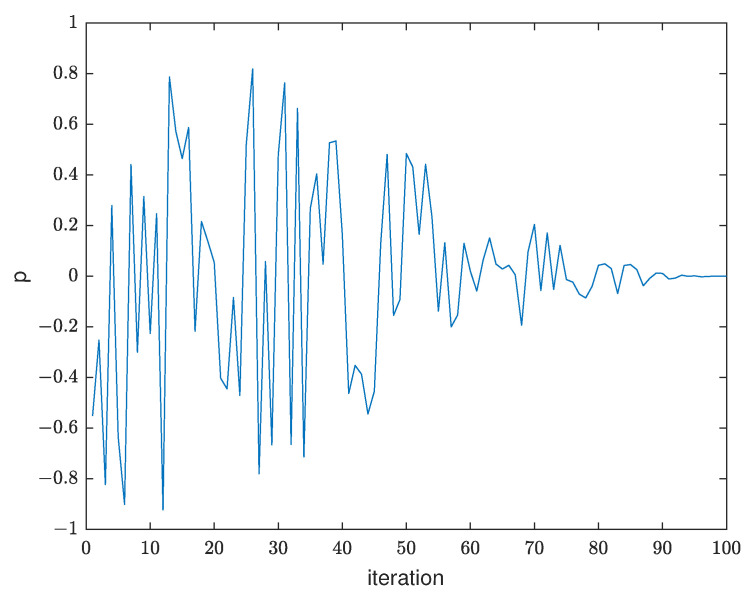
The change trend of *p*.

According to the theoretical idea of RPSO, appropriate perturbation can effectively improve the search ability of the algorithm. The addition of perturbation terms can significantly enhance the randomness of the algorithm in the early evolutionary stage which can better search the solution space. The existence of perturbation can avoid particles from falling into the local optimal solution and converging prematurely in the early stage. As the iteration proceeds, the perturbation item *p* gradually tends to 0 to ensure the convergence of the algorithm at the later stage. The detailed formula of *p* is described as follows.

(20) $$p=r_{2}\times (\left(\cos\left(t/T\times \pi \right)+1\right)/2$$

where $r_{2}$ takes a random number in the interval [−1, 1] and changes with iteration to ensure both forward and reverse perturbation exists. *t* is current iteration and *T* is the maximum iteration. The trend of *p*-value changes with iteration is shown in Figure 2.

Figure 2 illustrates the perturbation value *p* will converge to 0 with the iteration process, which meets the requirements of the setting rule of perturbation. During the iteration process, *p* takes positive or negative randomly in Figure 2 which improves the search ability of the algorithm.

## 4. Experimental Section

In this section, a series of tests involving feature selection problems is conducted to evaluate the performance the proposed approach. The problem definition of feature selection, along with the corresponding fitness function employed, is initially introduced in this section. Subsequently, the experimental setup and a comprehensive overview of the obtained results are presented. Finally, a detailed analysis of the convergence and stability of our proposed approach is conducted.

### 4.1. Problem Definition

Feature selection is a typical NP-hard combinatorial optimization problem. It is a process to choose the important feature subset in the original set of features [35]. Assuming that the dataset has *n* features, it may exist $2^{n}$ potential feature subset solutions. Furthermore, feature selection is to select the important subset from $2^{n}$ feature subset which can provide high classification accuracy. However, the number of selected features is also important.

### 4.2. Fitness Function

The fitness function plays important an role in PSO-based wrapper technique for feature selection. Classification accuracy and the number of selected features are two important valuation indicators. Therefore, the fitness function used in this paper includes the classification error rate and the number of selected features. The specific definition of the function is described as follows.

(21) $$\;\mathrm{fitness}\;=\alpha \times \;\mathrm{error}\;+\left(1-\alpha \right)\frac{SF}{TF}$$

where α controls the importance of classification quality and takes a number in [0, 1] interval. error represents the classification error rate obtained by the used classifier. *S*F and TF are the numbers of selected features and total features, respectively.

### 4.3. Experimental Set

A total of 15 datasets from the UCI machine learning repository [36] are used to test the effectiveness of BRPSO. Details about these datasets are shown in Table 1. The k-nearest-neighbor (kNN) classifier with *k* = 5 [37] is adopted as the classification model to test the quality of the feature subset. Furthermore, the n-fold cross-validation (with *n* = 10) is used on training data. α in fitness function is set to 0.99 to ensure classification accuracy.

The original BPSO [3], NBPSO [8], LIWBPSO [15], and BGWOPSO [38] are selected to benchmark with BRPSO to test the effectiveness of BRPSO. Referring to the existing literature [39], the population size and the maximum number of iterations are set to 20 and 100, respectively. The problem dimension is equal to the number of features in the dataset. Each algorithm independently runs 20 times to test the stability of algorithms. All peer algorithms are implemented by MATLAB-R2021b and tested on a computer with an AMD Ryzen 7 4800H CPU @ 2.90 GHz and 16.0G GB of RAM. The operating system running on this computer is Windows 10 (×64).

### 4.4. Experiment Results

#### 4.4.1. Numerical Comparison Results

The performance results of all algorithms were statistically tested in this work, utilizing T-test and Friedman-test to test the performance of each method, respectively. The T-test confidence interval is set to 95%. The symbol “+” indicates that BRPSO outperforms other algorithms, “−” indicates that BRPSO performs worse than other algorithms, and “=” indicates that BRPSO and the other algorithms perform comparably.

The results obtained by BRPSO on several benchmark datasets were compared with the average classification results of peer algorithms, as shown in Table 2. From Table 2, it can be seen that the results produced by BRPSO can achieve the best average classification accuracy among the 5 algorithms on 11 datasets, thus showing the superiority of BRPSO in feature selection for accurate classification. In addition, T-test results further clearly demonstrate the performance comparison results of BRPSO with other algorithms. It can be clearly seen that BRPSO achieves better results than BGWOPSO on all datasets. Furthermore, BRPSO achieves better classification accuracy than NBPSO, LIWPSO, and BPSO on more than half of the datasets.

In addition, we also compared the number of features selected by all algorithms, as shown in Table 3. From Table 3, it can be seen that BRPSO can achieve better classification accuracy with the smallest number of features that can be selected on 13 datasets. On two datasets with more than 200 features, namely LSVT Voice Rehabilitation, and arrhythmia, the other algorithms need to select more than 100 features to achieve better accuracy. In contrast, BRPSO only needs to select less than 100 features to achieve better results. This indicates that BRPSO is able to select features that are more conducive to classification accuracy. In addition, on datasets with more classification categories, such as arrhythmia and LibrasMovemen, which have 16 and 15 categories, respectively, BRPSO can select the smallest number of features to achieve the best classification accuracy. This indicates the superiority of BRPSO in dealing with complex classification problems.

To compare the comprehensive performance of all algorithms, the fitness of all algorithms was also calculated according to Equation (21). The results are shown in Table 4. From Table 4, it can be seen that BRPSO achieved the best comprehensive performance on 11 data sets. Although BRPSO did not perform as well as LIWPSO on BreastCancer, SPECT, Tic-Tac-Toe Endgame, and BreastEW, BRPSO was able to obtain better classification accuracy or select fewer features than LIWPSO on these datasets. From the t-test results, BRPSO obtained better performance than BGWOPSO on all datasets. In addition, better fitness values than BPSO, NBPSO, and LIWPSO are also obtained on more than half of the datasets.

#### 4.4.2. Statistical Test Results

In addition, we also summarize the statistical tests for all the experimental results. A summary of the results of T-test results for each strategy in Table 2, Table 3 and Table 4 is done to make an overall comparison of the performance of all algorithms. The comprehensive performance values in Table 5 are “+” minus “−” for each metric. Table 6 shows the results of the Friedman test for all algorithms on error, select, and fitness. Where error is 1 minus accuracy.

The results in Table 5 show that all the CP results of BRPSO outperform the other algorithms. In particular, the CP values compared with BGWOPSO show that BGWOPSO did not perform better than BPSO on any of the datasets. BRPSO also clearly performs better on more evaluation metrics on multiple datasets compared to the other three algorithms.

In this part, the Friedman test is utilized to demonstrate the comprehensive performance of the 5 peer algorithms. The Friedman test necessitates rating *K* algorithms on each dataset based on their absolute value. The best performing algorithm is ranked 1, while the worst performing algorithm is ranked *K*. As can be seen from Table 6, BRPSO achieves the optimal performance on all three evaluation metrics, further demonstrating the superior performance of BRPSO in solving the feature selection problem.

### 4.5. Convergence and Stability Analysis

**Figure 3:**
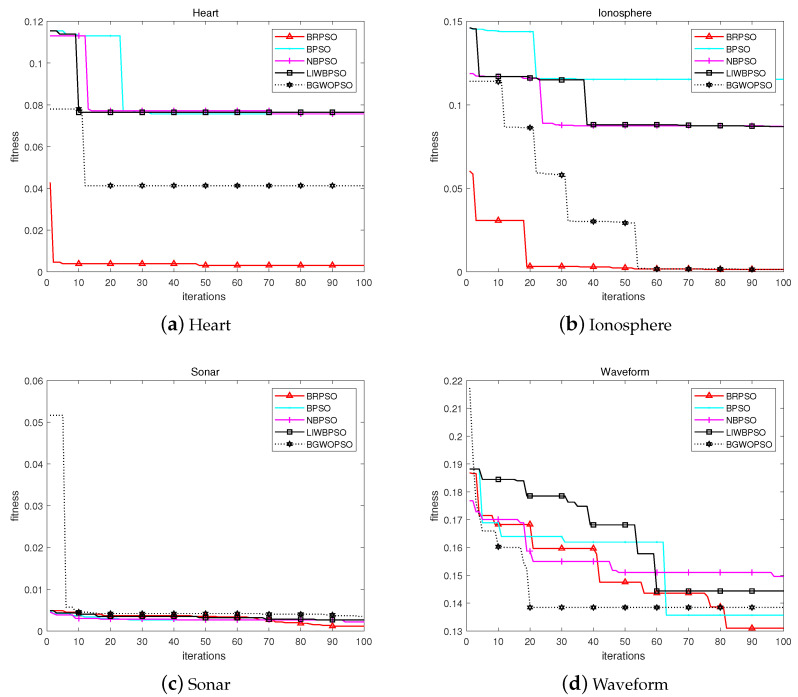

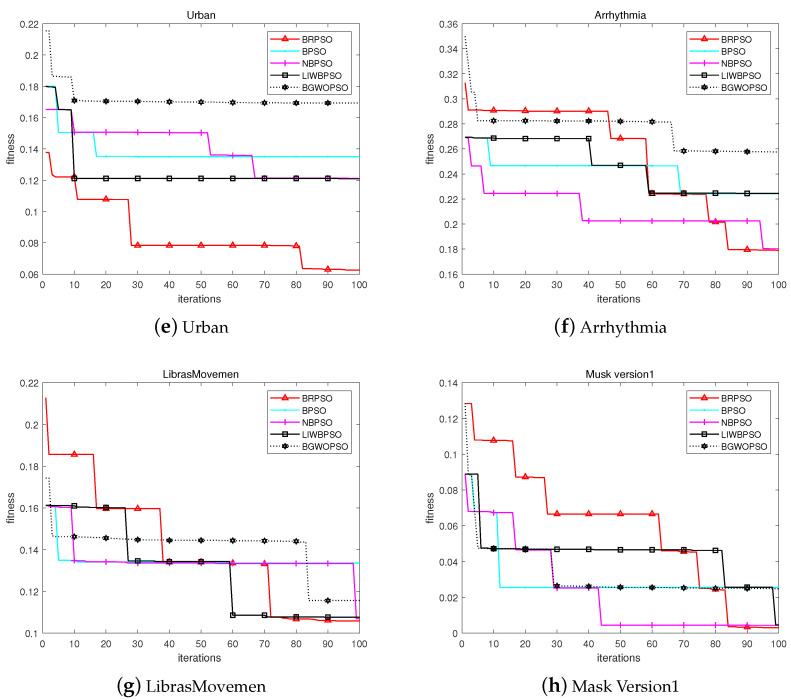
Convergence graph of each algorithm.

The convergence figures among five peer algorithms in eight datasets are shown in Figure 3. It can be found that BRPSO exhibits fast convergence speed on Heart, Ionosphere and Sonar datasets. In other five datasets, BRPSO displayed strong capacity in jumping out the local optimal solution and reached the best fitness value. The comprehensive capacity of BRPSO in convergence achieved favorite performance as shown in Figure 3.

**Figure 4:**
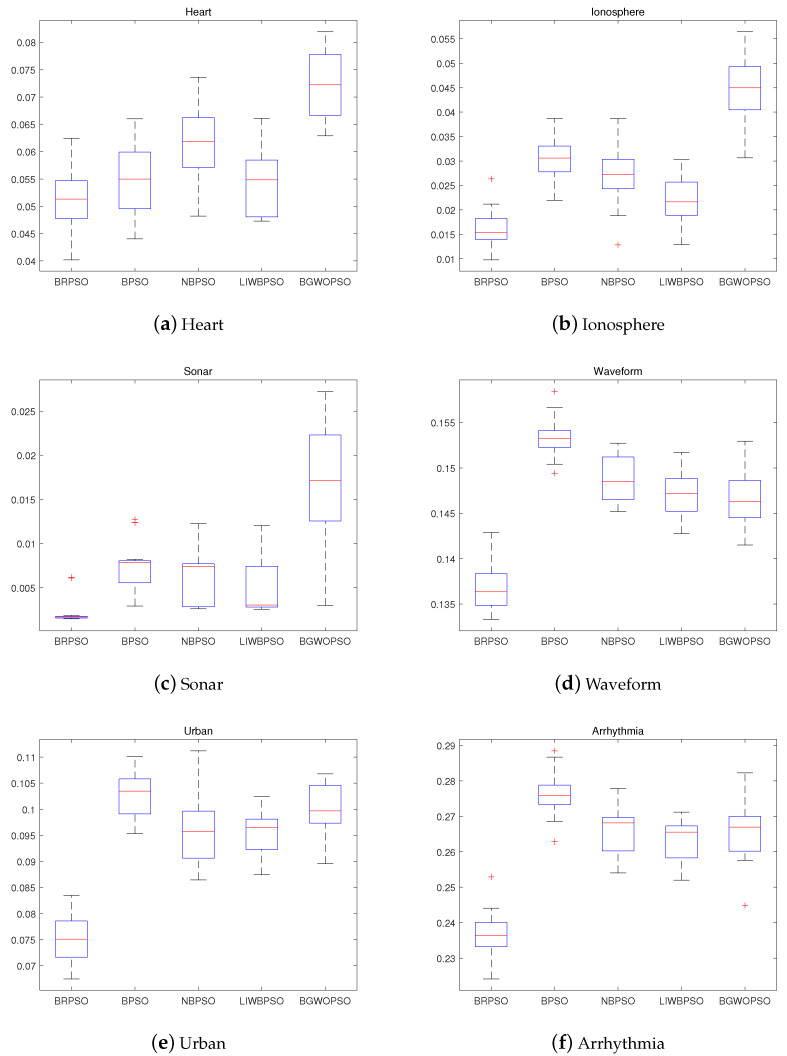

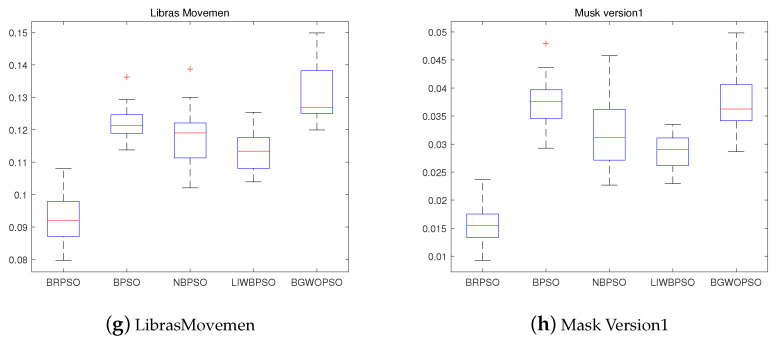
Boxplot of each algorithm.

The boxplot is used in this subsection to reveal the stability of five test algorithms. The boxplot which includes part of the results among five benchmark algorithms in 20 running times is displayed in Figure 4. The results shown in Figure 4 demonstrate that BRPSO obtained the high-quality best solution sets. As seen from Figure 4, the results gained by BRPSO in 20 running times are approaching which approved the stability of BRPSO.

## 5. Conclusions

This paper introduces a binary variant of Restructuring Particle Swarm Optimization (RPSO) specifically designed for discrete binary optimization problems. The proposed approach, known as Binary RPSO (BRPSO), eliminates the velocity updating formula and transfer function employed in Binary Particle Swarm Optimization (BPSO), resulting in a reduction in parameters and computational cost. BRPSO leverages historical experience from individual particles and the swarm to ensure its convergence capabilities. Furthermore, the integration of a decreasing perturbation factor effectively balances the exploration and exploitation abilities of BRPSO. To assess the performance of BRPSO, a comprehensive set of comparison experiments is conducted to solve feature selection problems. The experimental results, obtained by evaluating BRPSO against four benchmark algorithms across fifteen datasets, demonstrate its superior performance.

It is important to note that while our approach serves as a general algorithm for binary optimization problems, its performance may not outperform task-specific algorithms in certain scenarios. Nonetheless, this limitation also ensures the scalability of BRPSO for larger or more complex scenarios when combined with specific strategies. In future research, we anticipate potential modifications to the learning model and disturbance settings to address the unique challenges presented by real-world problems. Particular attention will be given to striking the right balance between exploration and exploitation capabilities, aligning with the demands of diverse problem domains.

## Figures and Tables

**Table 1:** Datasets description.

Dataset	No. Attributes	No. Instance	No. Class
BreastCancer	9	699	2
Heart	14	270	2
ionosphere	34	351	2
Sonar	60	208	2
SPECT	22	267	2
Waveform	41	500	3
Tic-Tac-Toe Endgame	9	958	2
Hill	100	1212	2
Urban	147	675	9
Vehicle	18	846	4
Musk version 1	168	476	2
LSVT Voice Rehabilitation	309	126	2
BreastEW	30	596	2
arrhythmia	279	452	16
LibrasMovemen	90	360	15

**Table 2:** Results on accuracy.

	BRPSO	BPSO	NBPSO	LIWPSO	BGWOPSO
BreastCancer	98.7	98.8(=)	98.7(=)	98.8(−)	98.5(+)
Heart	95.2	94.9(=)	94.1(+)	94.9(=)	93.1(+)
ionosphere	98.5	97.2(+)	97.5(+)	97.9(+)	95.8(+)
Sonar	100	99.6(+)	99.6(+)	99.7(+)	98.6(+)
SPECT	92.5	92.2(=)	91.9(+)	92.8(−)	90.7(+)
Waveform	86.7	85.1(+)	85.5(+)	85.7(+)	85.9(+)
Tic-Tac-Toe Endgame	84.2	84.6(−)	83.2(+)	84.6(−)	83.4(+)
Hill	70.8	64.5(+)	65.9(+)	65.7(+)	67.6(+)
Urban	92.8	90.1(+)	90.7(+)	90.8(+)	90.4(+)
Vehicle	82.1	81.7(=)	81.3(+)	82.0(=)	80.3(+)
Musk version 1	98.7	96.7(+)	97.2(+)	97.5(+)	96.7(+)
LSVT Voice Rehabilitation	97.4	96.5(+)	96.5(+)	97.0(=)	95.3(+)
BreastEW	99.6	99.5(=)	99.5(=)	99.7(=)	99.2(+)
arrythmia	76.5	72.6(+)	73.6(+)	73.8(+)	73.6(+)
LibrasMovemen	90.9	88.1(+)	88.5(+)	88.9(+)	87.1(+)

**Table 3:** Results on select.

	BRPSO	BPSO	NBPSO	LIWPSO	BGWOPSO
BreastCancer	2.975	3.175(+)	3.16(+)	3.18(+)	3.615(+)
Heart	4.815	4.8(=)	4.865(=)	4.78(=)	5.795(+)
ionosphere	4.425	7.935(+)	6.39(+)	5.985(+)	9.15(+)
Sonar	9.68	18.985(+)	16.5(+)	16.625(+)	18.75(+)
SPECT	7.04	7.555(+)	7.365(+)	7.235(=)	8.605(+)
Waveform	21.19	21.745(+)	21.79(+)	21.565(=)	27.63(+)
Tic-Tac-Toe Endgame	7.515	7.695(=)	6.76(−)	7.645(=)	7.31(=)
Hill	39.155	44.72(+)	43.55(+)	43.465(+)	57.86(+)
Urban	48.34	65.23(+)	62.14(+)	62.595(+)	67.59(+)
Vehicle	8.51	8.805(=)	8.685(+)	8.555(=)	10.45(+)
Musk version1	48.285	73.22(+)	69.075(+)	69.245(+)	66.78(+)
LSVT Voice Rehabilitation	64.98	119.315(+)	107.325(+)	108.115(+)	107.91(+)
BreastEW	4.145	6.36(+)	5.58(+)	5.205(+)	7.575(+)
arrhythmia	94.925	125.975(+)	120.635(+)	123.41(+)	122.575(+)
LibrasMovemen	21.8	34.79(+)	31.245(+)	31.9(+)	31.84(+)

**Table 4:** Results on fitness.

	BRPSO	BPSO	NBPSO	LIWPSO	BGWOPSO
BreastCancer	1.58 $\times \;10^{-2}$	1.51 $\times \;10^{-2}$(=)	1.63 $\times \;10^{-2}$(=)	1.50 $\times \;10^{-2}$(=)	1.86 $\times \;10^{-2}$(+)
Heart	5.08 $\times \;10^{-2}$	5.47 $\times \;10^{-2}$(=)	6.17 $\times \;10^{-2}$(+)	5.45 $\times \;10^{-2}$(=)	7.25 $\times \;10^{-2}$(+)
ionosphere	1.64 $\times \;10^{-2}$	3.00 $\times \;10^{-2}$(+)	2.71 $\times \;10^{-2}$(+)	2.24 $\times \;10^{-2}$(+)	4.42 $\times \;10^{-2}$(+)
Sonar	2.08 $\times \;10^{-3}$	7.42 $\times \;10^{-3}$(+)	6.32 $\times \;10^{-3}$(+)	5.36 $\times \;10^{-3}$(+)	1.67 $\times \;10^{-2}$(+)
SPECT	7.76 $\times \;10^{-2}$	8.11 $\times \;10^{-2}$(=)	8.34 $\times \;10^{-2}$(+)	7.42 $\times \;10^{-2}$(=)	9.63 $\times \;10^{-2}$(+)
Waveform	1.37 $\times \;10^{-1}$	1.53 $\times \;10^{-1}$(+)	1.49 $\times \;10^{-1}$(+)	1.47 $\times \;10^{-1}$(+)	1.47 $\times \;10^{-1}$(+)
Tic-Tac-Toe Endgame	1.65 $\times \;10^{-1}$	1.61 $\times \;10^{-1}$(−)	1.74 $\times \;10^{-1}$(+)	1.61 $\times \;10^{-1}$(−)	1.73 $\times \;10^{-1}$(+)
Hill	2.93 $\times \;10^{-1}$	3.56 $\times \;10^{-1}$(+)	3.42 $\times \;10^{-1}$(+)	3.44 $\times \;10^{-1}$(+)	3.26 $\times \;10^{-1}$(+)
Urban	7.48 $\times \;10^{-2}$	1.03 $\times \;10^{-1}$(+)	9.62 $\times \;10^{-2}$(+)	9.53 $\times \;10^{-2}$(+)	1.00 $\times \;10^{-1}$(+)
Vehicle	1.82 $\times \;10^{-1}$	1.86 $\times \;10^{-1}$(+)	1.90 $\times \;10^{-1}$(+)	1.83 $\times \;10^{-1}$(=)	2.00 $\times \;10^{-1}$(+)
Musk version1	1.62 $\times \;10^{-2}$	3.68 $\times \;10^{-2}$(+)	3.15 $\times \;10^{-2}$(+)	2.88 $\times \;10^{-2}$(+)	3.71 $\times \;10^{-2}$(+)
LSVT Voice Rehabilitation	2.79 $\times \;10^{-2}$	3.81 $\times \;10^{-2}$(+)	3.81 $\times \;10^{-2}$(+)	3.35 $\times \;10^{-2}$(+)	4.98 $\times \;10^{-2}$(+)
BreastEW	5.47 $\times \;10^{-3}$	6.64 $\times \;10^{-3}$(+)	6.82 $\times \;10^{-3}$(+)	5.12 $\times \;10^{-3}$(=)	1.01 $\times \;10^{-2}$(+)
arrhythmia	2.36 $\times \;10^{-1}$	2.76 $\times \;10^{-1}$(+)	2.66 $\times \;10^{-1}$(+)	2.63 $\times \;10^{-1}$ 1(+)	2.66 $\times \;10^{-1}$(+)
LibrasMovemen	9.28 $\times \;10^{-2}$	1.22 $\times \;10^{-1}$(+)	1.18 $\times \;10^{-1}$(+)	1.14 $\times \;10^{-1}$(+)	1.32 $\times \;10^{-1}$(+)

**Table 5:** Statistical results of T-test between BRPSO and other 4 competitors.

BRPSO vs.		BPSO	NBPSO	LIWPSO	BGWOPSO
(#)+	accuracy	9	13	8	15
	select	12	13	10	14
	fitness	11	14	10	15
(#)−	accuracy	1	0	3	0
	select	0	1	0	0
	fitness	1	0	1	0
CP		30	39	24	44

**Table 6:** Statistical results of Friedman test between BRPSO and other 4 competitors.

Rank	Error		Select		Fitness	
1	BRSPO	1.40	BRSPO	1.27	BRSPO	1.40
2	LIWPSO	1.93	NBPSO	2.6	LIWPSO	1.93
3	BPSO	3.67	LIWPSO	2.87	NBPSO	3.60
4	NBPSO	3.67	BPSO	4.13	BPSO	3.73
5	BGWOPSO	4.33	BGWOPSO	4.13	BGWOPSO	4.33

## Data Availability

The data presented in this study are available on request from the corresponding author.
